# Arrhythmogenic Cardiomyopathy: Molecular Insights for Improved Therapeutic Design

**DOI:** 10.3390/jcdd7020021

**Published:** 2020-05-26

**Authors:** Tyler L. Stevens, Michael J. Wallace, Mona El Refaey, Jason D. Roberts, Sara N. Koenig, Peter J. Mohler

**Affiliations:** 1 Departments of Physiology and Cell Biology and Internal Medicine, Division of Cardiovascular Medicine, The Ohio State University College of Medicine and Wexner Medical Center, Columbus, OH 43210, USA; tyler.stevens@osumc.edu (T.L.S.); michael.wallace@osumc.edu (M.J.W.); mona.elrefaey@osumc.edu (M.E.R.); sara.koenig@osumc.edu (S.N.K.); 2 Dorothy M. Davis Heart and Lung Research Institute, The Ohio State University Wexner Medical Center, Columbus, OH 43210, USA; 3 Section of Cardiac Electrophysiology, Division of Cardiology, Department of Medicine, Western University London, London, ON N6A 5A5, Canada; jason.roberts@lhsc.on.ca

**Keywords:** arrhythmogenic cardiomyopathy, desmosome, genetic diseases, sudden cardiac death

## Abstract

Arrhythmogenic cardiomyopathy (ACM) is an inherited disorder characterized by structural and electrical cardiac abnormalities, including myocardial fibro-fatty replacement. Its pathological ventricular substrate predisposes subjects to an increased risk of sudden cardiac death (SCD). ACM is a notorious cause of SCD in young athletes, and exercise has been documented to accelerate its progression. Although the genetic culprits are not exclusively limited to the intercalated disc, the majority of ACM-linked variants reside within desmosomal genes and are transmitted via Mendelian inheritance patterns; however, penetrance is highly variable. Its natural history features an initial “concealed phase” that results in patients being vulnerable to malignant arrhythmias prior to the onset of structural changes. Lack of effective therapies that target its pathophysiology renders management of patients challenging due to its progressive nature, and has highlighted a critical need to improve our understanding of its underlying mechanistic basis. In vitro and in vivo studies have begun to unravel the molecular consequences associated with disease causing variants, including altered Wnt/β-catenin signaling. Characterization of ACM mouse models has facilitated the evaluation of new therapeutic approaches. Improved molecular insight into the condition promises to usher in novel forms of therapy that will lead to improved care at the clinical bedside.

## 1. Introduction: Arrhythmogenic Cardiomyopathy

Arrhythmogenic cardiomyopathy (ACM) is a rare form of heart disease characterized by fibro-fatty replacement of ventricular myocardium [1]. ACM prevalence varies considerably by geographic location with a range that extends from 1:2000 to 1:5000 people worldwide. Italy has one of the highest prevalences of the condition, and males are more commonly affected at a ratio of 3:1 [2,3,4]. Studies have shown that ACM is responsible for 3–10% of sudden cardiac deaths (SCD) worldwide among individuals less than 40 years of age, with studies in Italy attributing over 20% of SCD cases to ACM in young athletes [4,5,6]. Although the risk of SCD is exacerbated by exercise, many malignant arrhythmic ACM events are not exercise related [7,8].

Average age of onset of the disease is approximately 30 years of age. Disease onset prior to adolescence is relatively rare, and most individuals destined to manifest a positive phenotype will do so prior to 65 years of age [9]. Electrical abnormalities generally precede structural defects, commonly referred to as the “concealed phase” [10,11]. ACM families are initially identified via an SCD event in 7–23% of cases [7], with some studies estimating SCD as the first clinical manifestation of ACM in 50% of probands [12,13]. Encompassing cardiomyopathies associated with a high risk of ventricular arrhythmias, ACM is a general term that includes multiple subtypes. The most common subtype of ACM is arrhythmogenic right ventricular cardiomyopathy (ARVC); however, left and biventricular dominant forms of the disease are well documented [3,14,15,16], including lamin types A/C (*LMNA*) and phospholamban (*PLN*) cardiomyopathies [17,18]. Notably, autopsy reports have suggested that as many as 76% of ACM cases possess left ventricular (LV) abnormalities [16,19].

Due to the absence of a single test that can confirm the condition, its diagnosis can be challenging. This has necessitated the development of task force criteria in an effort to standardize ACM diagnosis. Originally introduced in 1994 and subsequently modified in 2010, the task force criteria are a composite of clinical, structural, electrocardiographic, and genetic features that allow for clinical cases to be categorized as definite, borderline, and possible [1]. Following an ACM diagnosis, clinical management most often consists of some degree of exercise restriction and β-blockade. Among ACM patients deemed to be at significant risk for SCD, insertion of an implantable cardioverter device (ICD) should be considered. Among patients that experience ventricular arrhythmias, anti-arrhythmic drug therapy and catheter ablation are relied upon to suppress further episodes and avoid painful ICD shocks [20,21,22,23].

This review focuses on our current knowledge of the genetics underlying ACM and the mechanisms leading to disease onset and progression. Current disease models of ACM and environmental factors shown to contribute to disease development and progression will also be discussed, followed by a review of recent studies examining promising future therapeutics.

## 2. Genetics and Animal Models of Arrhythmogenic Cardiomyopathy

An underlying genetic culprit is identified in approximately 30–60% of ACM cases [11,24,25]. Although viewed as a monogenic disorder, 30–40% of ACM cases are sporadic, suggesting oligogenic and environmental contributions [11,24,25]. Notably, de novo and somatic mutations are not anticipated to account for significant proportions of sporadic cases given findings from a large study involving 209 genotype positive ACM probands, in whom only 1.4% had de novo variants [26]. Males are more likely to develop ACM and develop a more severe arrhythmogenic phenotype at an early age [9,25], possibly secondary to hormonal differences, as high testosterone levels are linked to more severe arrhythmogenic events [27]. The primary mode of transmission of desmosomal variants is considered autosomal dominant; however, autosomal recessive forms of ACM have been documented, most notably the cardio-cutaneous forms, including Naxos disease (*JUP*) and Carvajal syndrome (*DSP*) [28]. While autosomal dominant inheritance is the primary method of transmission, penetrance is quite variable in patients. Additional undiscovered genetic variants are likely attributed strongly to disease penetrance and the formation of genetic subtypes [29].

Putative genetic ACM culprits are quite diverse, as highlighted by the presence of 16 potential disease-causing genes (Table 1). ACM is commonly referred to as a disease of the desmosome and, indeed, reports document that 85–90% of all ACM-linked variants reside within desmosomal genes [26,30]. Outside the desmosome, additional ACM-linked genes encode proteins that are associated with the intercalated disc (ID), including α-T-catenin (*CTNNA3*), desmin (*DES*), N-cadherin (*CDH2*), tight junction protein-1 (*TJP1*), voltage-gated sodium channel alpha subunit 5 (*SCN5A*), and transmembrane protein 43 (*TMEM43*) [24,31,32]. Although the full array of genetic variants have been reported in ACM, the majority are truncating. One report indicated that 83% were nonsense, frameshift, or splice site mutations, while 14% were missense variants [3]. While the genetic cause, subtype, and incomplete penetrance results in significant variance in disease characteristics between individuals, features including fatty infiltration and increased prevalence of ventricular arrhythmias are common among ACM cases [29]. While the pathophysiology of ACM, particularly for fatty infiltration, is not completely understood, alterations to adipogenic pathways including the Wnt/β-catenin signaling and the Hippo pathway are commonly reported [33,34,35].

**Figure 1:**
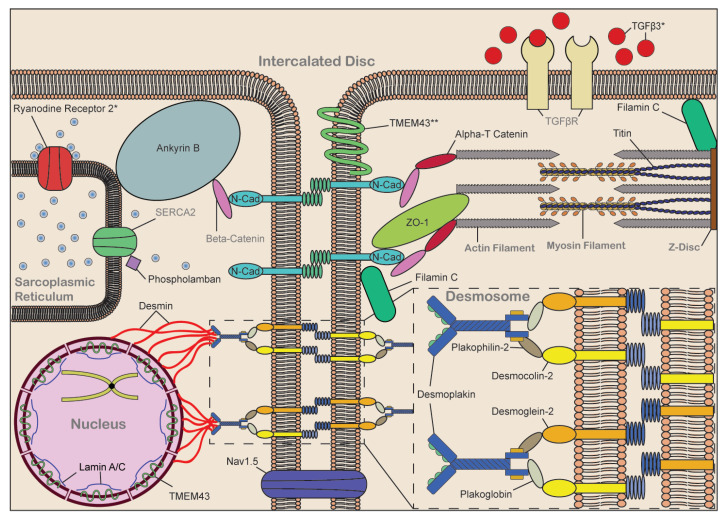
Schematic of ACM-associated proteins. Schematic of adjacent cardiomyocytes with key organelles and protein complexes labeled in grey bold text. Localization of the ACM-linked proteins provided, written in black text. Zoom in of the desmosome provided. * RyR2 and TGFβ3 considered borderline ACM genes. ** Conflicting results over TMEM43 localization.

Although identification of an underlying genetic culprit for ACM can be helpful for guiding proper clinical diagnosis and treatment, several variables make this challenging. Incomplete penetrance is commonly seen among familial cases of ACM. Multiple disease-causing variants have been identified in ACM, with variants in select genetic subtypes, such as TMEM43-p.S358L, *PLN*, *LMNA*, and filamin-C (*FLNC*), posing additional challenges due to association with severe or unique forms of ACM [24,50,60,61,62]. In addition, recent reports have suggested desmoplakin (DSP) related myopathies may be distinct from other ACM cases [63]. One of the most difficult challenges is determining if variants in these ACM-associated genes are disease causing or incidental noise. Within the ARVC database, the majority of variants listed (~70%) are classified as unknown or no known pathogenesis [36,37] (Supplementary Table S1). Figure 1 shows the localization of known ACM-associated proteins within cardiomyocytes.

### 2.1. Desmosomal Genes

**Figure 2:**
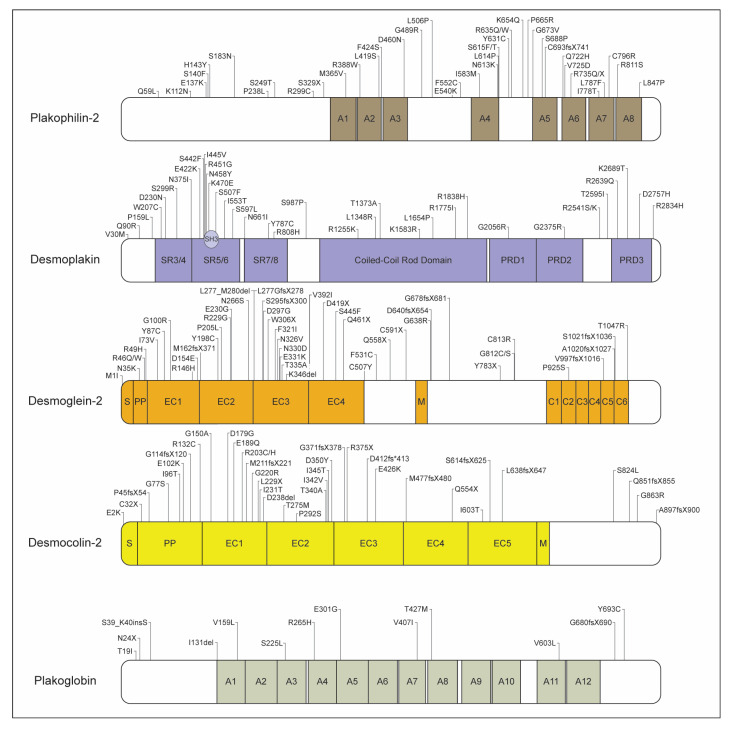
ACM-linked variant distribution among desmosomal genes. Linear diagram of the 5 desmosomal genes with labeled domains and the distribution of pathogenic ACM-variants. Variants of plakophilin-2 and desmoplakin limited to those mentioned throughout the review and missense variants among the ARVC database. Domain abbreviations: A, arm domain; SR, spectrin repeat; PRD, plakin repeat domain; S, signal peptide; PP, propeptide; EC, extracellular cadherin; M, membrane; C, cytoplasmic repeat.

The desmosome is one of three major protein complexes that comprises the cardiac ID, where it links desmin between adjacent cardiomyocytes and acts as a cellular anchor to maintain cardiac tissue integrity following force generation [64] (Figure 1). The first gene linked to ACM was the desmosomal gene junctional plakoglobin (*JUP*) [65]. Desmosomes also play a key role in intracellular signaling and gap junction function [7,64]. Associated genes plakophilin-2 (*PKP2*), *DSP*, desmoglein-2 (*DSG2*), desmcolin-2 (*DSC2*), and *JUP* comprise the majority of known ACM-causing variants, which are present in nearly 90% of cases with an identified genetic cause [7,31,32]. Desmosomal variants from the ARVC database are listed in Figure 2.

#### 2.1.1. Plakophilin-2

##### Genetics

Variants in PKP2 are the most common cause of ACM, accounting for 32–81% of ACM cases with an identified causative variant [25,26,30,38,39,40,41]. ACM-linked PKP2 variants frequently appear in highly conserved regions of the protein when compared to PKP2 variants in control populations [39]. Outside of its central structural role, PKP2 has been suggested to have a key role in recruitment of other ID proteins and may regulate expression of a variety of cardiac genes such as calcium handling genes, including ankyrin-B (ANK2), ryanodine receptor-2 (RYR2), and calsequestrin-2 [66,67]. Additionally, PKP2 has been shown to interact with PKC [68] and β-catenin [69], both suggested players in fibrosis and adipogenesis. Most disease-causing PKP2 variants result in expression of a truncated protein secondary to frameshift, nonsense, and splice site mutations [36]. Fortunately, PKP2 variant carriers are spared from cardiac–cutaneous disease because PKP2 is not expressed in stratified epithelium [70]. Generally, PKP2 linked ACM cases have a traditional ARVC like phenotype with minimal LV involvement, although mild LV dilation and dysfunction has been noted in later stages [7,24,25,66]. Insight into disease development secondary to PKP2 variants is gradually emerging. Characterization of a pathogenic variant, Q62K, revealed increased protein turnover, along with an inability to initiate desmosome formation [71]. Evaluation of the pathogenic variant C796R revealed decreased protein stability, mislocalization to the cytoplasm, and disrupted binding with DSP. The mechanism of increased protein turnover in this variant, along with additional pathogenic missense/frameshift variants (S615F, K654Q, and C693fsX741), was shown to be via increased calpain protease-mediated degradation [72].

##### Animal Models

With the majority of ACM-linked variants within desmosomal genes, the majority of ACM animal models have been designed to evaluate alterations in these genes. Whole body plakophilin-2 knockout (KO) mice were developed and were embryonically lethal at approximately embryonic day 11.5 (E11.5). Cardiac evaluation at E10.75 revealed multiple cardiac abnormalities, including atrial thinning, abnormal blood distribution, reduced ventricular trabeculation, abnormal ID structure with decreased adherens junction (AJ) heterogeneity, and disrupted DSP and DSG2 localization, resulting in DSP aggregate formation. Heterozygous (Het) KO mice were born healthy with no apparent cardiac phenotype [73]; however, further characterization revealed altered ID structure with sporadic or absent desmosomes, along with sodium current dysfunction [74].

A cardiac-specific and inducible (tamoxifen) KO mouse model was developed, aiming to evaluate the consequences at adult stages. Increased fibrosis, impaired wall movement, and dilation were observed in the right and left ventricle at 21 and 28 days post injection, respectively, and no animals survived 50 days post-injection [66]. This inducible model has a strong ACM phenotype with LV involvement, showing many traits of the human phenotype, including a concealed phase with altered connexin-43 (Cx43) and RyR2 function [67]. In addition, models with transgenic expression of truncated PKP2 (S329X, R735X) also result in cardiac remodeling that is exacerbated following exercise [75,76]. Truncated PKP2 has shown expression in cardiac tissue, and increased transgenic dose is correlated with increased severity of disease progression. Overall, loss of full length PKP2 results in major cardiac defects leading to ACM.

#### 2.1.2. Desmoplakin

##### Genetics

As a member of the plakin family, DSP directly interacts with the intermediate filament desmin to link the cytoskeleton to the remainder of the desmosome [64,77]. DSP is essential for recruitment of plakoglobin (PKG) and DSG for proper desmosome formation [78] and is involved with trafficking the key gap junction protein Cx43 [77]. Loss of DSP leads to decreased Cx43 at the ID, as well as increased Cx43 degradation caused by increased phosphorylation levels via RAS signaling [77,79], a key kinase pathway essential for proper cellular proliferation and differentiation found to be improperly regulated in many diseases. This ultimately results in slowed conduction velocity that may be a substrate for ventricular arrhythmias (VA) [80]. *DSP* is ubiquitously expressed in desmosomal containing cells, and consequently, pathogenic variants are associated with cutaneous/cardiac–cutaneous disorders, including woolly hair disease, palmoplantar keratoderma, and Carvajal syndrome. Variants in *DSP* result in a variety of cardiac phenotypes, including left, right, and biventricular dominant ACM and dilated cardiomyopathy (DCM), and they are associated with LV dysfunction, heart failure, and SCD [81].

A variant “hot spot” has been identified in an evolutionary conserved SH3 domain surrounded by multiple spectrin repeats in the N-terminal portion of the protein [39,82]. Molecular dynamic simulations show these structures are important for mechano-stabilization that prevents rupture upon force generation, shown to be disrupted by multiple pathogenic variants. Similar to variants within PKP2, select DSP N-terminal variants display increased sensitivity to calpain [83], while others variants show an inability to interact with PKG [84]. Outside the N-terminal region, DSP variants are thought to be pathogenic through loss of proper homodimerization or disrupted interactions with desmin.

##### Animal Models

Whole body KO of DSP also shows embryonic lethality at ~E6–E6.5 due to defects in desmosome rich extra-embryonic tissues, resulting in a failure of egg cylinder formation within the ectoderm [85]. Cardiac specific KO of DSP was analyzed via Cre-recombinase expression under an MLC2v promotor (activated ~E9 [86]). Mice were born at normal Mendelian ratios, but showed 50% lethality by two months of age. Fibro-fatty infiltration was apparent, and through use of lineage labeling, showed adipose tissue of both cardiac and non-cardiac origin [87]. Interestingly, although Cx43, Cx40, DSC2, DSG2, and PKP2 displayed altered expression or localization, PKG was unaltered [87] despite being commonly altered in ACM cases [88,89].

Two additional groups have generated het KO models using a αMHC-Cre system. Homozygous KO mice typically experienced embryonic lethality, although some survived until approximately two weeks of age with small hearts and unorganized chambers, with the presence of adipocyte-like cells. Het KO mice displayed a classic ACM like phenotype with altered Wnt/β-catenin signaling, fibro-fatty infiltration, moderate ventricular dilation with reduced ejection fraction (EF%), and more frequent ventricular arrhythmias with or without exogenous stressors [89]. Years later, Gomes et al. examined a similar haploinsufficient model with a similar phenotype, with exception to a lack of functional changes identified via echocardiography [80]. Interestingly, consistent with the mouse model, data from human hearts with ACM secondary to pathogenic *DSP* variants showed reduced PKG, Na_V_1.5, and Cx43 from the ID, and aggregates of Cx43 were also noted. Yang et al. established multiple transgenic variant models of DSP, identifying embryonic lethality in two variants (V30M, Q90R), and ID remodeling via disrupted desmin interactions [84].

#### 2.1.3. Desmoglein-2

##### Genetics

Two extracellular cadherin-like proteins, DSG2 and DSC2, stabilize the desmosome between adjacent cardiomyocytes. Along with *PKP2* and *DSP*, *DSG2* variants are found in a significant amount of ACM patients [25,26,30,38,39,40,41]. *DSG2* variants occur in evolutionary conserved residues and cluster at an N-terminal mutational “hot spot” containing the first three N-terminal cadherin domains, along with a prosequence region [39]. These extracellular cadherin (EC) domains are vital for dimerization and mechanically stabilizing desmosomes, which have been shown to be disrupted by variants D154E and V392I [90]. Outside of the cadherin repeats, a noncoding variant in the 5′ untranslated region (UTR), −317G > A, results in a loss of binding affinity with transcription factors. Evaluation of human tissue and ECG recordings revealed a borderline ARVC phenotype in patients with this noncoding variant [91]. N-terminal variants often prevent proper DSG2 processing before protein maturation [81]. Pathogenic variants within the intracellular portion of DSG2 can potentially dissociate interactions with PKG [81]. Although the majority of *DSG2* variants are autosomal dominant, gene dosage is likely important, as cases of homozygous or multiple disease causing variants have been identified, often associated with a more severe phenotype [60,92,93,94]. Homozygous variant F531C results in biventricular ACM with impaired ID ultra-structures [95]. Overall, variants in *DSG2* appear to generate a more severe form of ACM with more severe ventricular dysfunction, associated with a higher risk of heart failure in comparison to PKP2-linked ACM [96].

##### Animal Models

Complete KO of DSG2 has been analyzed by multiple groups. Removal of exons 7–8 in previous mouse models resulted in complete embryonic lethality [97], while models with a lack of exons 4 and 5 were capable of surviving with increased rates of lethality [98]. These mice had low expression levels of a truncated protein capable of ID localization. DSG2 KO mice displayed increased ventricular volume, diastolic dysfunction, reduced EF%, and fibrotic lesions. ECG traces following norepinephrine stimulation resulted in ventricular and atrial arrhythmias [98]. Increased ID heterogeneity and decreased desmosome formation was also observed [99]. Chelko et al. treated these mice with the glycogen synthase kinase-3β (GSK3β) inhibitor, SB-216763, and reported an improved cardiac function, decreased fibrosis, and normalized Cx43/PKG localization [100]. Overall, this model has an extreme, although variable, phenotype with many ACM features, including fibro-fatty infiltration and ID structural abnormalities.

A transgenic overexpression (αMHC) model of the ACM linked DSG2 variant N271S (N266S in humans) was generated and displayed 30% lethality by 3.6 weeks. A severe biventricular phenotype was observed with chamber dilation and reduced systolic function at 12 weeks, along with signs of ventricular wall thinning and increased necrosis. Conduction velocity was delayed along with multiple ventricular and atrial arrhythmias by six weeks. Fibrosis and calcification were noted as well; however, fatty infiltration was never observed [101]. Additional characterization in a later study showed reduced action potential (AP) upstroke velocity and sodium current, providing a unique potential arrhythmogenic mechanism associated with DSG2 variants [102].

#### 2.1.4. Desmocolin-2

##### Genetics

Although DSC2 structure and function is similar to that of DSG2, variants within DSC2 make up a smaller percentage of total ACM cases [25,26,30,38,39,40,41]. Like DSG2, multiple ACM linked variants cluster within DSC2′s N-terminus prosequence region at the N-terminus of the gene, shown to influence proper protein localization to the ID [81,103,104]. Multiple variants have been reported that impact the EC1 and EC2 domains, key for dimerization and stabilizing intramolecular interactions between adjacent EC domains [81]. Variants within the EC1 domain potentially destabilize DSC2 and its extracellular interactions [103,105,106]. Multiple variants within EC2 have also been reported, including a D350Y variant that alters interactions with calcium, key for stabilizing EC3 interactions [81,94,107]. Select variants have also been shown to cause mislocalization of DSC2 to the cytoplasm (E102K, I345T). In addition, multiple variants have been reported to alter binding affinity to PKP2/PKG, including truncated or non-expressed proteins (L229X, G371fsX378), loss of binding residues (A897fsX900), or improper protein maturation via impaired proteolytic processing (R203C, T275M) [108]. Outside of the traditional ARVC phenotype, LV and bi-ventricular dominant forms have been reported.

##### Animal Models

DSC2 overexpression in mice resulted in enlarged hearts with biventricular dysfunction, patchy areas of fibrosis, necrosis, and calcification by nine weeks of age, and prolonged QRS/QTc interval [109]. This was linked to extreme desmosome dysfunction, partially observed in fibrotic regions. While not representative of any known ACM-linked variants, overexpression of DSC2 did generate a rapid ACM-like phenotype producing focal regions of disrupted myocardium [109]. A unique Zebrafish model was generated based on a splice acceptor mutation (c.631-2A→G) resulting in a premature stop codon that led to decreased desmosome stability and a traditional ARVC phenotype caused DSC2 haploinsufficiency [110].

#### 2.1.5. Junctional Plakoglobin

##### Genetics

Variants in *JUP* account for the smallest number of ACM cases among desmosomal genes. Similar to PKP2, PKG is an armadillo protein that is key for linking DSP to DSG2 and DSC2. Despite structural similarities, the binding domains that PKG and PKP2 use to interact with DSP are not identical [78,111], possibly explaining the discrepancy in ACM cases between the two armadillo proteins. Additionally known as γ-catenin, PKG plays a pivotal role in both desmosome and AJ function [64], and protein recruitment (i.e., Cx43) [112].

Despite the rarity of ACM-associated *JUP* variants, alterations of PKG expression and localization are commonly seen in ACM cases and are believed to influence Wnt/β-catenin signaling, where increased nuclear PKG leads to decreased canonical signaling [33,88,89,113,114,115]. Due to structural and functional similarities, γ-catenin is capable of competing with β-catenin and ultimately results in suppression of canonical Wnt/β-catenin signaling [64,89]. Suppression of canonical Wnt/β-catenin signaling has been shown to promote adipogenesis, fibrosis, and apoptosis, and is believed to be one of the major drivers of fibro-fatty infiltration in ACM [64].

##### Animal Models

Multiple groups have established mouse models focusing on PKG alterations. Ruiz et al. developed a PKG KO mouse model that was lethal around E12–E16. Desmosomal and ID structural abnormalities, along with DSP mislocalization, were seen as early as E8, resulting in reduced contractility and tachycardia at E11.5 [116]. To avoid embryonic lethality, an inducible cardiac specific KO (αMHC/MerCreMer) with tamoxifen injections at six to eight weeks of age was developed. An ACM-like phenotype manifested at five months post injection with regional myocyte loss and fibrosis, along with right ventricular (RV) wall thinning, dilation, and increased cardiomyocyte size. Despite a lack of an arrhythmogenic phenotype, ID disorganization and increased SCD rates were observed as early as three months post injection [34].

Li et al. successfully generated a non-lethal KO model using αMHC-Cre system. A similar, more rapid overt cardiac phenotype was observed, including dilation, wall thinning, and fibrosis without fatty infiltration. This mouse model displayed SCD as early as one month, with an average lifespan of 4.6 months. Uniquely, electrical abnormalities including ventricular arrhythmias and conduction velocity defects were observed at baseline that were exacerbated after induction [117]. This phenotype was more severe when generated in conjunction with a β-catenin KO model [118].

The PKG 2157del2 variant, associated with both ARVC and Naxos disease, was also studied in murine models [65]. Transgenic overexpression of either (wild type) WT or the truncated protein (shown to mislocalize to the cytoplasm and nucleus) resulted in decreased LV fractional shortening, diastolic dysfunction, electrical abnormalities, and fibro-fatty infiltration, with a more severe phenotype in the truncated model [100,119]. Treatment of PKG 2157del2 mice with the GSK3β inhibitor, SB-216763, resulted in an improved cardiac phenotype, as seen in DSG2 KO mice [100].

### 2.2. Non-Desmosomal Genes

Despite the frequency of variants in desmosomal genes, variants among genes outside the desmosome still contribute to a significant portion of ACM cases. Many of these additional genes are within the ID or interact with the desmosome; however, additional nuclear, sarcoplasmic reticulum (SR), and calcium handling proteins have also been implicated. Variants in the UTR region of transforming growth factor 3 (*TGF3β*) were associated with an ACM phenotype in one study [120]; however, minimal studies since initial correlation make it unclear if *TGF3β* is an ACM causing gene. Variants in *RYR2* are known to be a common cause of catecholaminergic polymorphic ventricular tachycardia (CPVT), a primarily electrical cardiac defect [54]. However, structural remodeling and ACM cases caused by *RYR2* variants have been reported [54,121,122]. Outside of *TGF3β* and *RYR2*, multiple non-desmosomal genes have been connected to ACM.

#### 2.2.1. α-T-Catenin

α-T-catenin is responsible for cellular adhesion within the ID via interactions with N-cadherin (N-Cad), PKP2, and β-catenin, and is an uncommon cause of ACM with only two reported disease-causing variants at highly conserved residues (V94D and L765del). Each variant has been suggested to cause a traditional ARVC-like phenotype with mild LV involvement. The V94D variant results in abnormal localization to the cytoplasm, along with reduced binding affinity for β-catenin and PKG. In contrast, the L765del variant protein has increased homodimerization with either wild type (WT) or variant α-T-catenin, resulting in aggresome formation [42], a common defect seen in variants of other non-desmosomal proteins linked to ACM [17,44,45,123].

#### 2.2.2. Desmin

DES is the cytoskeletal intermediate filament that interacts with the desmosome via desmoplakin interactions, and is responsible for cellular organization and connections to other protein complexes, including the Z disc [44]. Cardiac disease caused by *DES* variants are often reported with skeletal muscle involvement, referred to as desmin-related myopathy (DRM) or desminopathy [43,44,124]. Variants associated with DRM or ACM result in altered desmin filament formation and localization, forming aggregates that can disrupt mechanical/chemical signaling and protein interactions [44,45,124,125,126]. Desmin variants have been reported to result in severe right-sided heart failure, and biventricular forms of disease have also been observed [44,45,125,127,128]. These variants are diverse in their localization within *DES*, ranging from the head domain (S13F), to multiple central rod domains (N116S, N342D). It remains unknown how different variants result in separate cardiac disorders, along with how families with a single variant display significant phenotypic variability.

#### 2.2.3. Lamin A/C

Variants in lamin A/C cause a unique LMNA cardiomyopathy, a subtype of ACM with significant LV involvement, and are one of the more common non-desmosomal causes of ACM [40,129]. Lamins are ubiquitously expressed intermediate filament proteins that form scaffolding structures around the nuclear periphery, key for gene regulation, genomic stability, and nuclear integrity [130]. Multiple diseases have been linked to LMNA due its ubiquitous expression, including ACM and DCM (independent or co-segregates with non-cardiac abnormalities), muscular dystrophy, peripheral neuropathy, and lipodystrophy [40,130]. Many variants commonly occur in conserved residues of the central rod domain (R190W, R72C, and G382V) or globular head of the protein (R644C), all of which are predicted to impact protein structure from in silico analysis [40], with select variants resulting in aggregate formation [17]. *LMNA* based ACM cases compared to desmosomal cases have shown significantly higher instances of bradycardia, a potentially unique feature to other forms of ACM [46].

#### 2.2.4. Phospholamban

Variants in the non-desmosomal protein PLN makes up a considerable amount of ACM cases among non desmosomal genes [30]. Key for proper regulation of cellular calcium, PLN acts as the natural inhibitor of sarco/endoplasmic reticulum Ca^2+^-ATPase (SERCA), the SR protein responsible for SR calcium reuptake following contraction [18]. Variants in PLN often result in phospholamban cardiomyopathy, a distinct form of cardiomyopathy with SERCA2 dysfunction that has an overlap of clinical features of ACM and may be considered a form of ACM itself. Generally, PLN-based ACM cases occur at an older age, but have more frequent LV involvement and increased risk of heart failure [25]. Cardiac phenotypes associated with the common R14del variant show extreme variability, including DCM and ACM of biventricular dominance [131]. Further characterization of the R14del variant revealed a potential pathogenic mechanism via aggresome formation [123]. When combined with WT PLN, this R14del variant results in extreme inhibition of SERCA1 [132]. Additional PLN variants have been identified in ACM cases [36], but have yet to be characterized in sufficient detail.

A PLN-p.R14del mouse model was established due to its being a prominent cause of cardiomyopathy within the Netherlands [18,131,133]. These mice showed a DCM like phenotype with ventricular dilation, myocyte disarray, and fibrosis along with increased propensity for SCD [132]. Human studies show R14del ACM cases typically have an additional variant to drive an ACM phenotype as opposed to DCM, so additional variants or stressors may make this a useful unexplored ACM model.

#### 2.2.5. Transmembrane Protein 43

Reports have established the TMEM43-p.S358L variant as a rare cause of a highly penetrant form of ACM. Like LMNA, TMEM43 is associated with the internal nuclear membrane and plays a role in nuclear organization and stabilization via interactions with lamin and emerin. Controversy concerning TMEM43 function and localization in cardiac tissue remains, as reports have suggested expression is absent from the nucleus and instead locates to the ID [134]. TMEM43-p.S358L is the only variant associated with ACM, but with significant supporting evidence that it alters homodimerization and protein interactions [135,136] and results in a severe form of ACM that is frequently biventricular and confers a high risk of SCD. Catheter ablation is frequently ineffective at suppressing ventricular arrhythmias, and mainstays of therapy are β-blockade, exercise restriction, and ICD insertion. Unlike desmosomal forms of ACM, experts have advocated for primary prevention ICD insertion based on sex-specific age cutoffs, even in the absence of a positive clinical phenotype [136,137,138].

The TMEM43-p.S358L variant results in an extreme ACM phenotype in humans, and a cardiac specific (αMHC) mouse model expressing human TMEM43 S358L showed a similar severe phenotype [135]. This model displayed extremely high rates of SCD starting before 20 weeks, with nearly no survivors by 30 weeks. These mice were treated with a GSK3β inhibitor via overexpression of a CnAβ1 splice variant, resulting in downstream activation of AKT, leading to phosphorylation and inactivation of GSK3. Treatment resulted in extended survival and partially improved cardiac structure and function in S358L positive TMEM43 mice [135].

#### 2.2.6. Titin

As the largest protein in mammalian cells, titin (*TTN*) variants are very difficult to study, with the majority of variants resulting in TTN truncation. Variants within *TTN* are tightly correlated with DCM, and are also involved with muscular dystrophy, hypertrophic cardiomyopathy (HCM), and ACM, with the mechanisms of how variants result in a variety of phenotypes remains unknown [48,49,139]. Although primarily linked to DCM, specific genomic backgrounds or additional factors may promote an ACM phenotype to develop [49,140,141]. One study sequenced *TTN* in eight ACM families and identified seven rare *TTN* variants characterized by classic signs of ACM with LV involvement and fibro-fatty infiltration, one of which (T2896I) was evaluated in further detail due to complete segregation of the disease with the variant. This variant occurs inside an immunoglobin (IG) domain required for generating passive cardiomyocyte tension, unrelated to protein interactions [49]. Phenotypically, TTN-ACM cases are less severe compared to desmosomal based cases, but more so than ACM cases caused by non-desmosomal variants [48].

#### 2.2.7. Filamin C

*FLNC* is a structural protein responsible for linking the sarcomere to the plasma membrane and extracellular matrix in skeletal and cardiac muscle, and is involved in maintaining ID integrity [50,142,143]. Variants in *FLNC* have been shown to cause restrictive cardiomyopathy, DCM, and ACM with predominant LV involvement [50,52,144]. A recent study identified two ACM-linked variants in *FLNC* (59_62DLQRdel, K2260R), along with multiple null variants following genetic screening of ACM patients negative for variants in clinically examined genes. Further evaluation of tissue from SCD victims with *FLNC* null variants revealed reduced protein levels at the ID within the LV, but not the RV compared to controls. In addition Cx43 and DSP ID levels were reduced [50], potentially disrupting both ID stability and electrical signaling [77,79]. Interestingly, minimal changes to PKG and no alteration to GSK3β levels were examined, common features in classic ARVC [50]. Additional studies have shown similar unique trends in ID protein localization [51], suggesting disease development may be unique in FLNC cases compared to others.

#### 2.2.8. Voltage-Gated Sodium Channel Alpha Subunit 5

Na_V_1.5, transcribed via the *SCN5A* gene, is the primary voltage gated sodium channel in cardiomyocytes linked to a variety of arrhythmogenic disorders as well as DCM [53,145,146]. Similar to *TTN*, variants in *SCN5A* are more closely linked to DCM, but specific genomic backgrounds may promote ACM development [53,146,147]. Reports have suggested that PKP2 and Na_V_1.5 co-localize to the ID, and that *SCN5A* loss-of-function variants may contribute to a classic ARVC phenotype. A recent novel variant I137M was identified in an ACM patient with a variety of ventricular arrhythmias, although mild RV dysfunction and dilation were noted as well, which was not unexpected, as variants in ion channels have resulted in myocardial structural abnormalities. Additionally, another novel variant R1898H resulted in a similar phenotype with an identified decreased in sodium current [53]. Finally, a severe splice variant (C3840 + 1G > A) led to a loss of function of the Na_V_1.5 channel, resulting in a severe electrical dysfunction with mild RV dysfunction. Although fibrosis was noted, fatty tissue was not discovered, suggesting this may be a unique form of ACM or ACM-like cardiomyopathy [148].

#### 2.2.9. Tight Junction Protein 1/Zonula Occludens 1

Rare variants within *TJP1*, encoding tight junction protein-1 (TJP1), have recently been identified as a potential rare cause of ACM through whole exome sequencing [56]. TJP1, also known as ZO1, is a scaffolding protein that has been shown to interact with the ID through a variety of proteins, including Cx43, N-Cad, and α-T-catenin. Although an intriguing finding, further validation of TJP1 as a genetic culprit of ACM should likely be pursued prior to its incorporation of clinical genetic testing panels.

#### 2.2.10. N-Cadherin

The extracellular cadherin of the adherens junctions of the ID, N-cadherin, is vital for proper ID formation, stability, and protein recruitment to mechanically link cardiomyocytes via interactions with multiple catenins. Similar to DSG2 and DSC2, variants have been reported within the extracellular domains that are key for protein dimerization and cellular adhesion. One variant that causes a traditional ARVC phenotype with mild wall thinning and fibro-fatty infiltration, D407N, occurs in a highly conserved residue from humans to zebrafish and is predicted to be damaging via in silico analysis [57]. The Q229P variant, which strongly segregates within the affected family, was also predicted to be damaging and resulted in a similar phenotype, with the exception of lack of fibro-fatty infiltration [58].

#### 2.2.11. Ankyrin-B (ANK2)

A recent study connected *ANK2* to ACM. Ankyrin-B is responsible for the localization and stabilization of key ion channels, transporters, and ion exchangers to the cell membrane and t-tubules, such as the sodium–calcium exchanger (NCX) and sodium–potassium ATPase. Previously, variants in *ANK2* have been linked to a variety of arrhythmogenic disorders including ankyrin-B syndrome [149], atrial fibrillation [150], and sinus node disease [151]. The AnkB–ACM study evaluated a proband with an identified E1458G variant, revealing biventricular dysfunction, sustained VA, and baseline bradycardia. Additional families with a genotype-negative ARVC diagnosis were revaluated, and an additional M1988T AnkB variant was discovered that segregated within a family, although likely benign variants within DSG2 and DSC2 were identified as well. This family displayed a similar phenotype to AnkB cardiac specific KO mice, with reduced AnkB and NCX expression, along with abnormal Z-line targeting.

The cardiac specific KO of ankyrin-B in mice exhibited both electrical and structural cardiac abnormalities. This mouse model displayed baseline ECG abnormalities at baseline (bradycardia, QT prolongation) and developed sustained VA following epinephrine stimulation, resulting in multiple cases of sudden death. Although adipogenesis was not observed in this model, biventricular dilation with wall thinning, biventricular systolic dysfunction, and widespread fibrosis were findings that mirrored those observed in other ACM models. Abnormal heterogeneous expression of β-catenin was also observed. GSK3β inhibition, when administered either before or after the presence of cardiac dysfunction, resulted in an improved phenotype similar to that of control mice [59].

### 2.3. Additional ACM Models

A unique mouse model of a mutant laminin receptor-1 (LAMR1) resulted in a severe ARVC phenotype that was identified by chance. This transgenic model was established from a retroposon insertion, resulting in a 13-amino acid mutation, causing severe fibrosis and calcification of the RV free wall, which never extended to the LV despite equal expression level. Altered gene expression via disrupted histone protein-1 interactions were observed [152]. Despite the lack of major electrical abnormalities, this model may be useful in identifying fundamental differences between the LV and RV, and evaluating why select protein variants have a more severe effect on one ventricle over another.

Zebrafish studies do not have the ideal physiological relevance to humans as mice do, but are still capable of producing unique ACM models. The previously mentioned zebrafish model of the DSC2 variant c.631-2A→G was able to reveal not only a reduced expression of DSC2, but disrupted desmosome and cardiac structure [110]. Additional zebrafish models examining alterations to PKG (2057del2) and DSP knockdown revealed multiple ACM phenotypic characteristics, partially restored following SB-216763 treatment [153,154].

Canine and feline models of ACM have also been studied, which might be a more accurate representation of the human disease. Basso et al. used a boxer dog model to examine ventricular arrhythmias using holter monitors, where 23 genetic candidates for ARVC were identified. All dogs displayed fatty/fibro-fatty infiltration within the RV, with RV dilation and LV lesions seen in about half these animals. Ventricular arrhythmias were common among these animals and SCD was reported in nine dogs [155]. Another study reexamined feline models with congestive heart failure of RV origin. A traditional ARVC phenotype was seen with RV and RA dilation, along with fibrosis, arrhythmic events and/or fatty infiltration seen in all cats, some of which affected the LV [156]. Although the genetic culprits for ACM in the boxer dog model have not been identified, the similar human like phenotype may make evolved mammals more effective for analysis of therapeutic approaches.

Although not as effective at studying organ level cardiac physiology or arrhythmias when compared to animal models, cell models have contributed significantly towards variant protein analysis and their biochemical properties [157]. Isolation of human-induced pluripotent stem cells (iPSCs) allows for quick and efficient analysis of genetic variants by differentiation of iPSCs into cardiomyocytes (iPSC-CMs) [157], and this has been utilized to form engineered heart tissue [83]. HL-1 atrial cells have been utilized for evaluation of the cellular consequences associated with ACM linked variants, as well as evaluating the consequences of gene silencing [72,89,157]. Desmosome containing cell lines, including buccal mucosa cells, have significant value for evaluating structural and adhesive consequences associated with desmosomal variants [157].

## 3. Environmental Factors

One of the hallmarks of ACM is incomplete penetrance, even among a family with the same genetic variant. Some carriers may be asymptomatic for most of their life, while others may experience SCD at a young age, making it clear that in addition to the genetic background of these patient, additional environmental factors play a major role in the progression of the disease, as seen in a study of two sisters with drastically different phenotypes despite having the same PKP2 variant. Studying twins with ACM has shown significant diversity in phenotypic development as well [158]. Exercise is understood to be a major driver of ACM progression, and patients are advised to restrict any physical activity to minimize the risk for VA/SCD, as well as to prevent further cardiac remodeling. Additional environmental factors that increase cardiac stress, such as emotions, habitat conditions, and pregnancy may also contribute towards phenotypic progression.

### 3.1. Exercise

Depending on the genetic background and current disease progression, treatment and therapeutic options may vary significantly between individuals. However, exercise restriction is highly recommended for all carriers of an ACM-linked variant, and is considered for variant carriers without a phenotype [20]. One study in Italy analyzing 1642 competitive athletes identified 6% as possessing ARVC. Strikingly, 25% of SCD cases among athletes have been deemed secondary to ARVC in Italy [159]. This overrepresentation of SCD, along with the majority of cases occurring during athletic activity, strongly suggests exercise is potentially detrimental to ACM/ARVC patients [159,160]. High cardiac strain has been linked to impaired myocyte cell–cell adhesion, resulting in premature cardiomyocyte death that may induce cardiac remodeling [20]. When ARVC patients or genotype positive individuals were studied for six years, a significant correlation was identified between exercise and both reduced RVEF% and increased rates of SCD [161]. Loss of cellular adhesion is a popular mechanism studied in many models of ACM [162], with multiple cell models showing reduced cell–cell adhesion following overexpression or reduction of desmosomal proteins [163,164]. Desmosomal dysfunction may be enhanced via volume overload and mechanical stress, providing additional evidence connecting ACM disease progression and vigorous exercise [4].

One study focused on exercise changes on ACM phenotype progression in patients with desmosomal variants. Of 87 studied individuals, endurance athletes were more likely to meet task force criteria (TFC) for ARVC diagnosis, and were more likely to develop ventricular tachycardia (VT) and ventricular flutter (VF), along with heart failure. Interestingly, only endurance athletes developed heart failure. These athletes were categorized by hours exercised per year, and those who exercised the most have the highest risk compared to more casual athletes. In fact, those who reduced exercise (≤515 h/year) showed improved survival following an initial sustained VT/VF event [165]. Finocchiaro et al. examined over 400 cases of SCD among athletes, including 48 cases of ACM. The majority (61%) of SCD cases occurred during exertion, including 91% of those diagnosed with ARVC. Interestingly, LV involvement was a common feature among ARVC athletes, with 35% of cases showing LV fibro-fatty infiltration and 85% with LV fibrosis [166].

Two ACM mouse models described previously, a PKG heterozygous KO [167] and truncated PKP2 (R735X) overexpression model [75], both underwent swim training to accelerate disease progression. Trained mice displayed an increased incidence of spontaneous VT at an earlier age than untrained mice and developed a more severe ACM phenotype [167]. Factors affecting oxygen availability and air quality, such as high elevation, high levels of pollution, or being underwater may enhance cardiac stress during exercise. In addition, extreme temperatures or high humidity can lead to increased workload of the myocardium compared to normal conditions while exercising [168].

Among non-athletes, sudden death occurs less frequently during exercise or exertion. Over 75% of cases of death resulted during daily activities at “rest”, including at home, work, or walking on the street, with only about 3.5% occurred during some sort of sport or exercise. The remaining percentage died during unique events not considered a normal daily activity, including surgery or car accidents, or childbirth [169]. Further complexity is added to the role of exercise among ACM development in genotype negative individuals, or those without a variant in a known ACM-associated gene. La Gerche et al. observed that athletes without genetic variants still developed an ACM phenotype with ventricular arrhythmias and systolic dysfunction. However, athletes without an identified mutation were less likely to develop severe RV dysfunction (33% to 3%) when compared to those with a mutation. Interestingly, no fibro-fatty infiltration was noted [170], further indicating a separate phenotype among those without a mutation. While it has been proposed that a separate exercise-induced RV cardiomyopathy may explain an ACM like phenotype with select differences, studies have challenged this, suggesting the phenotype is identical between the two groups [30].

### 3.2. Additional Factors

Beyond exercise, additional external factors are proposed to be potential drivers for either arrhythmias or disease progression through additional cardiac stress. Multiple emotional triggers, including anger and stress, may increase sympathetic drive via β-adrenergic signaling, causing increased oxygen demand to maintain increased myocardial contractility and heart rate [168,171]. Therefore, competitive sports, even those among low cardiac stress, could be a stimulus for arrhythmias and SCD. Indeed, analysis of SCD among athletes identified two individuals who experienced lethal arrhythmias while playing golf [166], categorized under the least cardiac strenuous section of exercise [168]. Multiple arrhythmogenic conditions have been previously associated with increased prevalence of arrhythmias following emotional distress, including atrial fibrillation, CPVT, and long QT syndrome. Among non-athletes with ACM, stressful situations including minor car accidents, falls without apparent injuries, car theft, or child birth have all been stimuli resulting in SCD (~20% of deaths among non-athlete ACM patients).

## 4. Clinical Management of Arrhythmogenic Cardiomyopathy

Although ARVC is most commonly an autosomal dominant, genetically inherited disease, only up to 50% of patients have an identifiable desmosomal variant, leaving the cause of remaining ACM cases due to less common or unknown variants [172]. ARVC should not be considered a monogenic disease, but rather an oligogenic or polygenic disease. Many ARVC associated alleles have low pathogenicity, and a family member harboring just one variant might not display the ARVC phenotype [173]. Xu et al. found digenic and compound heterozygous variants to compile a significant portion of ARVC probands [94], which emphasizes the potential role of the gene dosing effect of ARVC penetrance [173]. Further, incomplete penetrance creates variation in the severity of symptoms across a family, potentially complicating recognition of familial disease, particularly if thorough cascade screening is not performed [174]. Unfortunately, diagnosis of ACM remains challenging, resulting in SCD as the first symptom of the disease in some cases [175]. In 2010, updated task force criteria gave clinicians further guidance to diagnose the disease with testing modalities including 2D echocardiogram, cardiac magnetic resonance (CMR), and ECG measurements [1].

The most concerning clinical symptom that clinicians screen for is effort-induced syncope. Other common clinical findings upon testing include T-wave inversion in leads V1–V4 of the ECG, ventricular arrhythmias, such as isolated premature ventricular contractions and non-sustained ventricular tachycardia on Holter monitoring, and right ventricular dilation on imaging studies [174].

Although specific International Task Force guidelines are in place for proper ARVC diagnosis, limitations still exist. Over-diagnosis from genetic screening, misinterpretation of ECG and echocardiogram recordings, and underutilization of CMR can all lead to misdiagnosis, especially considering the symptom overlap with other cardiomyopathies [176]. Since publication of the updated task force guidelines, more advanced imaging techniques have become more prominent in diagnosis. With 3D echocardiogram, accurate measurements of RV volume and RVEF% can be made, providing a useful tool for RV systolic function quantification. Further, CMR with late gadolinium enhancement can help with ACM diagnosis when only the left ventricle is involved. CMR ultimately provides the best measurement for RV wall abnormalities, RV volumes, and RV-EF. CMR abnormalities alone are unusual unless the ACM variant resides in the left ventricle alone [177].

Depending on the severity of symptoms, among individuals that have a positive clinical phenotype, management usually starts with restriction of physical exercise and β-blockade [165]. ICD insertion is recommended for patients deemed at significant risk for SCD following appropriate risk stratification. Additional pharmacological intervention with anti-arrhythmic drugs may also be advised to minimize the incidence of sustained VAs, which can result in painful and distressing ICD shocks [178]. Similarly, catheter ablation can also be used to suppress VAs, and a combined endocardial–epicardial approach is often necessary, given that the disease originates in the epicardium and migrates inwards. Heart transplant is the last option and may be required for refractory ventricular arrhythmias or heart failure [174,179]. With the wide range of ACM symptoms and therapeutic options, treatment is best individualized through risk stratification tools and expert clinical care.

### 4.1. Risk Stratification for SCD and Implantable Cardioverter–Defibrillator (ICD) Placement

Prior to the initiation of treatment strategies for ACM patients, particularly ICD insertion, effective risk stratification is crucial. Clearly, the most concerning consequence of ACM is SCD. The best prevention method for known SCD risk is an ICD, so predictions for risk of SCD are essential in targeting patients that require ICD treatment [178]. Identifiable variables for proper risk stratification include sex, age, cardiac syncope events in the prior six months, frequency of non-sustained VT, frequency of premature ventricular contractions (PVCs) within 24 h, number of leads with T-wave inversions, and RVEF%. A recently developed risk calculator showed particular promise by providing VA risk projections across 528 definite ARVC patients with no history of sustained VA, which decreased inappropriate ICD implantation compared to the 2015 task force model. This updated model is currently awaiting additional validation with external, more diverse cohorts [180]. The more common model, the 2015 International Task Force Consensus Statement Risk Stratification Algorithm of ICD placement, uses similar variables and places patients into class indications based on risk factors. Class 1 patients have the greatest risk, and placement in this class requires prior VT or VF, severe RV dysfunction, or severe LV dysfunction. The greater the symptom, the higher the class indication for a patient. This model has been shown to work well clinically, but class 1 and class 2a patients had indistinguishable ventricular fibrillation/flutter events, and the model is limited in its ability to help in primary prevention [181]. As more information surfaces about left ventricular involvement in ACM, the ability of CMR to identify left ventricular subtypes may be critical in future risk stratification models. A 2019 publication by Miles and colleagues found that the vast majority of SCD cases in ACM patients had left ventricular involvement [15]. Beyond risk assessment from clinical phenotype, certain genotypes are also associated with an increased risk of future major arrhythmic events. ACM subtypes associated with PLN, LMNA, FLNC, TMEM43-pS358L, and DSP have unique features that have separate reported guidelines/suggestions for treatment options [63,176,178,182]. While the 2010 guidelines for ARVC diagnosis have been an improvement, they fail to properly evaluate the genetic background of the disease and account for ACM subtypes outside of ARVC, including left dominant forms [176]. Regardless of risk assessment, exercise restriction is a precautionary measure every ACM patient should take. Should an ACM patient be asymptomatic under proper therapy, noncompetitive and low strain activities are acceptable. Unfortunately, exercise restriction is not sufficient in isolation, as made evident by a study by Wang and colleagues, which showed 58% of athletes still experienced VA after severe reduction in exercise (>80%) [183].

Patients with high risk for SCD from risk stratification should swiftly receive ICD therapy, or if antiarrhythmic drugs and lifestyle changes fail to manage ARVC symptoms. In 2003, Corrado and colleagues evaluated the impact of ICD implantation on SCD prevention in 132 ARVC patients. About 48% of patients who received appropriate ICD intervention did not experience episodes of VT [184]. Males were shown to have a much higher survival rate (95% vs. 65% five-year survival rate) than controls when ICD was used as a primary therapy for cardiomyopathy management, but this same trend did not translate as strongly to females (97% vs. 85% five-year survival rate) [185]. Unfortunately, inappropriate ICD shocks can be discharged by sinus tachycardia or atrial tachycardia, which adds morbidity to the patient and poses a risk to cardiac health. Proper ICD intervention, however, can be further controlled with other therapeutic options like drug therapy or ablation [20].

### 4.2. Anti-Arrhythmic Medications

Antiarrhythmic medications represent a critical therapeutic approach for ARVC patients. After insertion of an ICD, many ARVC patients continue to require anti-arrhythmic medications to reduce the frequency of ICD shocks. Marcus et al. showed that amiodarone significantly decreased the risk of clinically relevant VA (ICD shock or sustained VT) in ARVC patients undergoing ICD therapy [23]. In another study, a combination of flecainide with sotalol/metoprolol was shown to terminate arrhythmias in six of eight patients with diverse ARVC symptoms and variants. Overall, flecainide with sotalol/metoprolol could be a combination therapy for patients refractory to a single-agent therapy or ablation [186], but larger studies are urgently needed. Importantly, a CAST (cardiac arrhythmia suppression trial) study showed higher incidence of death due to arrhythmia and shock in patients who previously suffered myocardial infarction and took flecainide compared to the placebo group, suggesting patients with ventricular scaring should avoid flecainide [187]. A randomized drug study with flecainide and ARVC patients is currently underway (Pilot Randomized Trial With Flecainide in ARVC Patients, NCT03685149, currently ongoing) to provide more insight into the efficacy of flecainide [188]. Overall, there are no published randomized trials of ARVC patients using various anti-arrhythmic drugs, so drug therapy is often left to the clinician’s discretion based on personal history. Importantly, there is no clear consensus on the best anti-arrhythmic drug; therefore, it likely depends on the individual patient.

### 4.3. Catheter Ablation

Catheter ablation is frequently used as a tertiary treatment option for ARVC patients after pharmaceutical intervention and ICDs. Anti-arrhythmic drugs are initially considered to reduce the frequency of VA to prevent painful ICD discharges. However, ablation procedures can often more effectively reduce the risk of recurrent arrhythmias. Although ablation procedures are considered palliative rather than curative, combined endocardial–epicardial ablation procedures have been shown to be remarkably effective [174]. Mahida and colleagues compared the effectiveness of anti-arrhythmic drugs (AAD) and beta blockers (BB) to VT ablation in ARVC patients with a minimum of three VT episodes. A single ablation procedure (both epicardial/endocardial and endocardial only) left 35% of patients free of VT for three years, while 28% of AAD/BB patients were VT free after three years, forcing many patients to require subsequent ablations. However, following a total of 75 patients after the last ablation, 71% of endocardial/epicardial ablation patients were VT-free after three years compared to 47% of patients who underwent endocardial ablation only [189]. Another study with an average follow-up of over four years reported a cumulative VT-free survival rate of 71% across 62 ARVC patients after endocardial only or epicardial/endocardial ablations, which again displayed the heightened success rate afforded by epicardial ablation [190]. In contrast, Christiansen and colleagues found that although ablation procedures decreased the burden of VA in ARVC patients, recurrence of arrhythmias was evident in 74% of cases just five years after the first ablation procedure [191]. The low percentage of patients that underwent epicardial ablation procedures (16%) may have contributed to the high recurrence rate, which again highlights the importance of epicardial ablation when necessary. Although not completely exempt from surgical complications or need for multiple procedures, catheter ablation remains a good therapeutic option after ICD and anti-arrhythmic drug treatment if ventricular arrhythmias still endure.

### 4.4. Sympathectomy

The autonomic nervous system has long been known to modulate heart rate and cardiac output via the sympathetic and parasympathetic nervous systems via innervation from the right/left stellate ganglia and medulla to the heart, respectively. Reducing sympathetic output via neuraxial modulation by left cardiac sympathetic denervation (CSD) (surgical dissection and resection of the stellate ganglion) protected against various refractory VA in five of nine patients with structural heart disease when all other therapies failed [192]. Preliminary ARVC-specific studies suggest bilateral CSD could be a valuable tool when anti-arrhythmic drugs, in combination with ICD and catheter ablation, fail to stop recurrent VT [193,194]. Although larger ARVC patient sample groups should be studied, ARVC CSD data agrees with more general studies showing the ability of bilateral CSD to decrease recurrent VT (and therefore ICD shock intervention) in patients with structural heart disease, particularly when drug therapy and epicardial/endocardial ablation have failed to treat the arrhythmias [195,196,197,198].

### 4.5. Heart Transplant

Heart transplant remains the last option for ARVC therapy. If refractory ventricular arrhythmias remain after exploring all other therapeutic options, or if the disease has progressed to left or right ventricular heart failure, or more rarely, diffuse biventricular heart failure, heart transplant is required [179]. Overall, survival rates in ARVC patients who received heart transplants are better compared to patients with other cardiomyopathies such as restrictive, ischemic, dilated, and hypertrophic over a follow up period of 10 years. Although severe heart failure in ARVC is rare, carriers for a *PKP*2 variant alongside a second ARVC variant were the most likely to suffer severe RV dysfunction and require heart transplant.

## 5. Future Implications

Successful management of ACM must address the three major components of the disease: myocardial damage (fibrosis and myocyte apoptosis), electrophysiological abnormalities (action potential remodeling and ventricular arrhythmias), and inflammation [199]. Garcia-Gras et al. previously identified that loss of desmoplakin resulted in a reduction of Wnt/β-catenin signaling [89]. SB-216763 was initially discovered to inhibit GSK-3β, therefore activating glycogen synthesis and expression of β-catenin [200]. Further, SB-216763 successfully rescued the ACM phenotype in a zebrafish model for ACM, which expressed a human 2057del2 variant in the plakoglobin (PKG) encoding gene. Interestingly, SB-216763 was also able to rescue and restore the reduction in *I_Na_* and *I_K1_* in the zebrafish model of ACM [153]. A second zebrafish model, deficient in desmoplakin by knockdown of *dspa* and *dspb* genes, was also rescued via pharmacological treatment with SB-216763 [154]. Even in rare forms of ACM (a rare *ANK2* variant), Wnt/β-catenin activation therapy prevented and partially reversed the development of the ACM phenotype in an AnkB cKO murine model [59]. The ability of Wnt/β-catenin pathway activation to rescue various forms of ACM from different gene variants suggests that the Wnt/β-catenin may be a common underlying pathway disrupted in ACM development. Future studies should explore other GSK-3β inhibitors as potential therapies for ACM to gain further insight into the specific mechanism of protection provided by GSK-3β inhibition, allowing these drug trials to move to clinical trials.

Gene therapy can allow for the mutant gene to be replaced, or potentially even repaired, and has been explored as a potential treatment for genetic muscular dystrophies and associated cardiomyopathies [201]. Although few studies have explored gene therapy for ARVC, gene therapy has been shown to delay onset of familial HCM if treatment begins shortly after birth in a transgenic mouse line. HCM is an autosomal dominant genetic disorder, like many forms of ACM, and is linked specifically to variants in the sarcomeric proteins, causing SCD in young adults [202]. In preliminary gene therapy research for ACM, the loss of cardiac Wnt signaling due to DSP deficiency was restored by genetic manipulation of DSP expression in a ACM type 8 zebrafish model [154], giving hope that future genetic intervention could reverse the ACM phenotype in well documented variants. While gene therapy shows great promise, the use of gene silencing may be problematic for many ACM cases, as many ACM associated genes, including desmosomal genes, do not show tolerance for haploinsufficiency [50,66,74,85].

In summary, ACM is an inherited disease that results from fibro-fatty remodeling of the myocardium and subsequently predisposes subjects to life-threatening ventricular arrhythmias. The complexity of this cardiac disorder is partially attributed to the diversity of genetic abnormalities. At the moment, there is no widely-available therapeutic option that targets the patho-physiologic course of the disease; rather, clinical management of ACM patients is restricted to the treatment of symptoms. Understanding the role of genetics and environmental factors that contribute to disease progression and its underlying pathophysiological mechanisms is crucial to developing new treatment strategies for this complex, life threatening disorder.

## Figures and Tables

**Table 1:** A list of all genes linked to ACM/ARVC. Provides information of the localization of each gene product in the cell, along with the count of ACM linked variants and associated phenotype [36,37].

Gene	Protein	Structure or Location	Pathogenic Variants *	ACM Prevalence **	Phenotype	References
*PKP2*	Plakophilin-2	ID/desmosome	171	23.4–57.6%	RV dominant ACM	[25,26,30,38,39,40,41]
*DSP*	Desmoplakin	ID/desmosome	86	1.6–15.7%	RV, LV, and biventricular ACM, DCM, Carvajal syndrome (recessive), palmoplantar keratoderma, additional cutaneous diseases.	[25,26,30,38,39,40,41]
*DSG2*	Desmoglein-2	ID/desmosome	50	4.0–20.4%	RV and biventricular ACM	[25,26,30,38,39,40,41]
*DSC2*	Desmocollin-2	ID/desmosome	42	1.0–8.3%	RV, LV, and biventricular ACM	[25,26,30,38,39,40,41]
*JUP*	Junction plakoglobin (PKG)	ID/desmosome	15	<1–3.0%	RV dominant ACM, Naxos disease (recessive), palmoplantar keratoderma, woolly hair disease	[25,30,39,41]
*CTNNA3*	α-T-catenin	ID/area composita	2	<2.6% (among ACM cases without common mutation)	RV dominant ACM	[42]
*DES*	Desmin	Intermediate filaments/cytoskeleton	11	<1–2.2%	RV and biventricular dominant ACM. DCM, HCM, DRM, muscular dystrophy	[41,43,44,45]
*LMNA*	Lamin A/C	Nuclear envelope	16	3.5–3.7%	RV and biventricular dominant ACM. DCM, HCM, muscular dystrophy	[40,41,46]
*PLN*	Phospholamban	Sarcoplasmic reticulum	4	2.2–12.4%	RV, LV, and biventricular dominant ACM, DCM	[18,25,26,30]
*TMEM43*	Transmembrane protein 43 (LUMA)	Nuclear envelope/ID	3	<1–2.2%	RV, LV, and biventricular dominant ACM, DCM, muscular dystrophy	[25,26,30,41,47]
*TTN*	Titin	Sarcomere	10	<1% (18.4% of ACM cases negative for variants in common genes)	RV and biventricular dominant ACM, DCM, HCM, muscular dystrophy	[48,49]
*FLNC*	Filamin C	Sarcomere/ID		<1% (7.5% of ACM cases negative for variants in common genes)	RV, LV, and biventricular dominant ACM, DCM, restrictive cardiomyopathy	[50,51,52]
*SCN5A*	Sodium channel Na_v_1.5	Cell membrane		<1–1.8%	RV dominant ACM, DCM, HCM, Brugada syndrome, long QT syndrome	[26,53,54]
*TJP1*	Zonula occludens 1 (ZO1)	ID/tight junction		<1% (<5% of ACM cases negative for variants in common genes)	RV, LV, and biventricular dominant ACM	[26,53,55,56]
*CDH2*	N-Cadherin	ID/adherens junction		<1% (2.7% of ACM cases negative for variants in common genes)	RV and biventricular ACM	[55,56,57,58]
*ANK2*	Ankyrin B (AnkB)	Z-lines/T-tubules		Unknown	RV dominant ACM, long QT-syndrome, atrial Fibrillation, ankyrin-B syndrome	[57,58,59]

* Variant count determined by those listed within the ARVC database [36,37]. ** Prevalence calculated by the percentage of ACM cases with an identified mutation in the gene of interest vs. total ACM cases). Abbreviations: RV, right ventricle; LV, left ventricle; ACM, arrhythmogenic cardiomyopathy; ARVC, arrhythmogenic right ventricular cardiomyopathy; DCM, dilated cardiomyopathy; HCM, hypertrophic cardiomyopathy; DRM, desmin related myopathy.

## Supplementary Materials

The following are available online at https://www.mdpi.com/2308-3425/7/2/21/s1, Table S1: Numerical distribution of ARVC variants from the ARVC database.
