# Supplementary material for: Arrhythmogenic Cardiomyopathy: Molecular Insights for Improved Therapeutic Design
Source: J Cardiovasc Dev Dis. 2020 May 26;7(2):21. doi: 10.3390/jcdd7020021 (PMC7345706; doi:10.3390/jcdd7020021)
Supplement: Supplementary file 1 [file jcdd-07-00021-s001.pdf]

Journal of
Cardiovascular Development and Disease

Supplementary Materials

Arrhythmogenic Cardiomyopathy: Molecular Insights for Improved Therapeutic Design

Tyler L. Stevens [1], Michael J. Wallace [1,2], Mona El Refaey [1,2], Jason D. Roberts [3], Sara N. Koenig [1] and Peter J. Mohler [1,2,*]

[1] Departments of Physiology and Cell Biology and Internal Medicine, Division of Cardiovascular Medicine, The Ohio State University College of Medicine and Wexner Medical Center, Columbus, OH, 43210, USA.; tyler.stevens@osumc.edu (T.L.S.); sara.koenig@osumc.edu (S.N.K.)

[2] Dorothy M. Davis Heart and Lung Research Institute, The Ohio State University Wexner Medical Center, Columbus, OH, 43210, USA; michael.wallace@osumc.edu (M.J.W.); mona.elrefaey@osumc (M.E.R.)

[3] Section of Cardiac Electrophysiology, Division of Cardiology, Department of Medicine, Western University London, N6A 5A5 Ontario, Canada; jason.roberts@lhsc.on.ca

* Correspondence: peter.mohler@osumc.edu; Tel.: +1-614-247-8610

Table S1. Numerical distribution of ARVC variants from the ARVC database [37,38]. Separated by mutational type and pathogenicity of mutation (no known and unknown classifications combined as unknown) and organized by gene. Does not include identified intronic or synonymous variants.

Gene	Missense Pathogenic	Missense Unknown	Indel/FS Pathogenic	Indel/FS Unknown	Nonsense Pathogenic	Nonsense Unknown	Splice Site Pathogenic	Splice Site Unknown	UTR Pathogenic	UTR Unknown
PKP2	38	63	67	3	39	0	27	1	0	2
DSP	33	107	19	10	29	2	5	1	0	4
DSG2	29	77	9	2	6	0	6	2	0	1
DSC2	23	38	9	4	4	0	6	0	0	3
JUP	10	15	3	0	1	0	1	3	0	2
CTNNA3	1	0	1	0	0	0	0	0	0	0
DES	7	10	2	0	1	0	1	1	0	0
LMNA	7	22	4	0	4	0	1	1	0	1
PLN	2	4	1	1	1	0	0	0	0	4
TGFB3	0	3	0	0	0	0	0	0	2	3
TMEM43	2	29	0	0	0	0	1	1	0	16
TTN	8	197	0	15	0	6	2	5	0	0
